# Clustering drives cooperation on reputation networks, all else fixed

**DOI:** 10.1098/rsos.230046

**Published:** 2023-04-26

**Authors:** Tamas David-Barrett

**Affiliations:** ^1^ Trinity College, University of Oxford, Oxford OX1 3BH, UK; ^2^ Population Studies Institute, Helsinki 00101, Finland

**Keywords:** social networks, evolution of cooperation, reputation, free-riding, clustering coefficient, microfoundations

## Abstract

Reputation-based cooperation on social networks offers a causal mechanism between graph properties and social trust. Using a simple model, this paper demonstrates the underlying mechanism in a way that is accessible to scientists not specializing in networks or mathematics. The paper shows that when the size and degree of the network is fixed (i.e. all graphs have the same number of agents, who all have the same number of connections), it is the clustering coefficient that drives differences in how cooperative social networks are.

## 1. Introduction

Early work on the evolutionary conundrum of costly cooperation focused on interactions that were dyadic in nature: the behaviour was seen as taking place between two entities. For instance, the question ‘why would Being A help Being B if it is costly for A and benefits only B?’ is dyadic as it concerns the interaction only between A and B. Even when one agent is a group, the question tends to be posed for interactions between an individual and a group: why would Being C give up something precious for group D, to which C may belong but so do many others? In this case as well, the interaction is dyadic, it is between C and D, even if the latter is an entity on a higher organizational level.

Between the 1960s and 1980s, two vastly different solutions to this puzzle were provided. First, the inclusive fitness mechanism showed that cooperation can emerge among close relatives based on shared genes [1–6]. Second, the reciprocal interaction mechanism showed that cooperation can emerge if agents have repeated interactions, can remember each other and can adjust their behaviour according to the past actions of the other [7–10]. This latter mechanism also had evolutionary foundations and is present in a wide range of species apart from humans [11].

Even though these two solutions were entirely different, one following the logic of biology, the other of economics, they were identical in that they both lacked interaction structure. Bacterium A helped bacterium B in a dyadic fashion, and hero C gave up its resources for the group D in a one-to-many way, also dyadic between individual and a ‘blob’ of a group. While the idea that interactions could form a network is present in these examples, the fact that the structure of the network could be important for the rise of cooperation is not in the focus. Structure had been ignored, because in a network in which the question is about *dyadic* cooperation, it can be interesting if one interacts with few or many, but it is irrelevant whether those others are also connected to each other and if so how.

Two insights changed the approach to cooperation, gradually emerging over the past 30 years.

First, the assumption that all interesting processes necessarily take place dyadically was altered by the anthropological, and also every-day, observation that people gossip [12–14]. When they do this, they pass on information about shared acquaintances, behind the acquaintance's back. Also for this, at least three agents are needed. Being A tells Being B that their shared connection C did something wrong, for instance, by adopting a cheating rather than a cooperative stance. Gossip speeds up the detection of cheaters, and thus it is a robust way of ensuring cooperation [15–23].

Second, network science, which emerged in interaction with, but following a different historical logic to the problem of cooperation, provided a method to structure reputation dynamics [24–26]. The network science approach highlighted the importance of the social network's structural properties and pointed out that the cooperation-enhancing effect of reputation increases with higher interconnectedness [24,27–30].

Thanks to the successful merger of these two traditions, the past 20 years have seen the emergence of a large literature that looks at the interaction between characteristics of a social network, in particular, the density and the degree distribution, and the space for costly cooperation to emerge [31–44]. Empirical findings from experiments on humans in several cultures have provided evidence that clustering alone does not, or at least does not necessarily, promote cooperation; it can, however, be a powerful driver when combined with a space for reputation formation [22,45–53], this is especially so when partner-selection itself is reputation based [54,55].

It is to this tradition that the current paper aims to offer a contribution. Recent papers suggested a causal link between macro-societal demographic processes and the social network's micro-structure, showing how falling fertility, urbanization and migration can reduce the propensity to cooperate among individuals [56–58]. These models relied on the literature's insights on the relationship between network structure and cooperation but lacked the instantiation for the case of *k*-regular *n*-sized connected graphs. The objective of this paper is to fill this gap.

## 2. Methods and results

The first part of methods introduces a game in which the agents meet repeatedly, but their interactions have no structure, and there is no possibility for gossip. The second part introduces graph structure and gossip into the same repeated game framework. The third part shows how the local interconnectedness, measured in the clustering coefficient, affects cooperation.

Note: the primary aim of this paper is to illustrate the mechanics of the relationship between the clustering coefficient and cooperation for scientists who are not themselves in the field of repeated games on networks. The models are particular instantiations of the literature's findings, which first appeared stated differently but with the same qualitative meaning, three decades ago [24–27], and have been supported by both theoretical and empirical evidence since [22,28,31–40,44–48,59–62]. Note that the special case to the general results illustrated here is the emergence of cooperation on grids in cellular automata, which were at the core of the rise of complexity science [63–65].

### 2.1. Everyone plays everyone, nobody gossips

Let us define a social group made of *n* agents. The agents play the dyadic Prisoner's Dilemma games where the pay-off matrix is symmetric, and is

$$p=\{\left\{\;p_{1,1},p_{1,2}\},\{\;p_{2,1},p_{2,2}\right\}\}.$$

Note: an implicit assumption in this model is that higher pay-off is the objective of every agent. This could be either in a biological setting in which pay-off increases evolutionary fitness, or—equally—in an economic setting in which higher pay-off leads to social or cultural success. Thus, within this model, having higher pay-off is an intrinsic motivation of the agents and does not have direct effect on the mechanics. That is, the agents aim to receive more pay-off, but the way they use the pay-off does not directly enter the model.

Let us assume that 0 < *m* < *n* players are randomly chosen as having type ‘cheater’, and thus *n*-*m* players have type ‘cooperator’.

Each player tracks every other player's type, where *d_i_*_,*j*_ is *i*'s agent's expectation of *j*'s type. Initially all agents assume that everyone else is a cooperator.

Agents are randomly paired and play their strategy the following way: if agent *i* expect agent *j* to be a cooperator, then agent *i* plays her strategy according to her type. If, however, she thinks that *j* will be a cheater, then she will cheat independent of her own type. In other words, if we expect the other to play nice, we will play nice if we are nice, and play dirty if we are naughty, but if we expect the other to play dirty, then we always play dirty even if we are nice. Formally:

{i,j}∼ U{1,…,n}|i≠j,

that is, we randomly pick the two agents. Then:

$$a_{i}=\{\begin{array}{ccc} \mathrm{cheat} & \mathrm{if} & d_{i,j}=\mathrm{cheater}\\ {\mathrm{type}}_{i} & \mathrm{if} & d_{i,j}=\mathrm{cooperator} \end{array},$$

where *a_i_* is the action of agent *i*.

After their interaction, the agents update their expectation of the other's type to the action played by the other (thus, in this first version of the model, beliefs are only based on action):

$$d_{i,j}=a_{j}$$

and

$$d_{\;j,i}=a_{i}.$$

Notice that because of the way the actions are chosen, if a partner sees you as cheater even just once, they will never trust you again.

**Figure 1.**
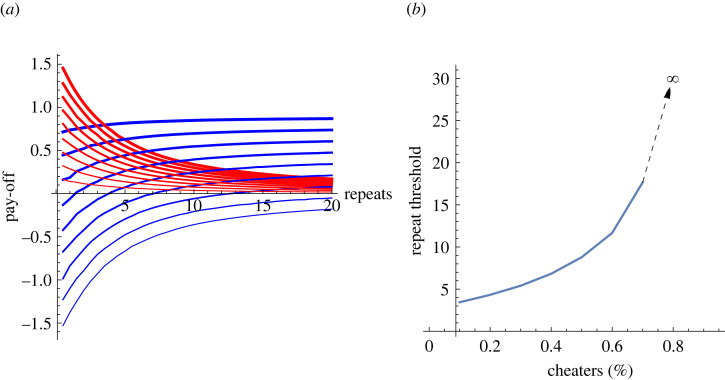
The interaction between repeat number, pay-offs and cooperation thresholds. (*a*) The cooperators' and cheaters' average pay-offs as a function of the number of repeated interactions per agent. Blue: cooperator, red: cheater. The thickness of the lines corresponds to the proportion of cooperators among the agents, with the thickest (topmost) lines corresponding the case when there is only a single cheater, i.e. *m* = 1, and the thinnest (bottommost) corresponding to the case when there is only a single cooperator, i.e. *m* = 9. (*b*) How many repeated interactions are needed for the cooperating strategy to have a higher pay-off than the cheating one, as a function of the proportion of cheaters. (Average of 1000 repeats. Note that the line always goes to infinite if *n*−*m* = 1 and the pay-offs are Prisoner Dilemma structure. That is, this model is not about the emergence of cooperation, only the maintenance. Pay-offs *p* = {{1,–1.6},{1.5,0}}, see the electronic supplementary material, SM1.) The figure illustrates that in the ‘plain vanilla’ case, the maintaining cooperation is easier when the interaction frequency increases, or the cheater ratio falls.

Let the interactions repeat until an average agent is in play *r* times. The repeat number, *r*, and the number of cheaters, *m*, drive the total pay-offs (figure 1*a*).

The results (figure 1*a*) show that:

(i) cheater pay-off decreases and cooperator pay-off increases as the number of interactions increase independent of the number of cheaters. (That is, the red lines are all going down, and the blue lines are all going up);
(ii) as the cheater number increases the pay-offs of both cheaters and cooperators increase. (The thinner lines are under the bolder ones, for both colours);
(iii) with the increasing cheater number, the pay-off drops faster for cooperators than cheaters. (The blue lines take a larger space than the red ones); and
(iv) as a consequence, the lines corresponding to the same number of cheaters (the red and blue lines of the same thickness) meet at different repeat numbers.

The crossing point of the red and the blue lines, given a particular number of cheaters, determines the repeat number above which it is advantageous to be a cooperator. The higher the cheater number, cooperation repeat threshold is also higher (figure 1*b*). Notice that the fact that the line in figure 1*b* is increasing means that once cooperation emerges, an evolutionary or learning mechanism would turn the entire group into cooperators.

### 2.2. Social network and gossip

Let us change the above ‘plain vanilla’ model by introducing a fixed interaction structure in terms of a network and allow gossip.

Let us assume that the *n* agents form a connected graph of degree *k*. (That is, each node, i.e. dot on the social network graph, has *k* ‘friends’.) I use this structure because it most closely resembles a human group in which *n* is magnitudes larger than *k*. Our species' groups tend to be so large that no individual can meaningfully be connected to every other group member, while the advantage of having more connections pushes people to have more connection if they can [66]. Thus, people tend to end up with a similar number of social connections.

Let us also assume that each time an agent is in an interaction, she decides her move based on what she thinks about the alter's type *and* what her trusted friends think, where her ‘trusted’ friends are those agents that are connected to her, and she still thinks that their type is cooperator. For this, let *e_i_*_,*j*_ denote the private expectations (belief) held by agent *i* about agent *j*. Notice that as the belief is formed partially by beliefs of other agents, the set {*e_i_*_,*j*_}*_j_* is the agent *j*'s reputation.

$$e_{i,j}=\{\begin{array}{ccc} \mathrm{cheater} & \mathrm{if} & d_{i,j}=\mathrm{cheater}\\ \mathrm{cheater} & \mathrm{if} & d_{i,j}=\mathrm{cooperator}\;\mathrm{and}\;\frac{\tilde{k}}{k+1}\geq 0.5\\ \mathrm{cooperator}\;\mathrm{otherwise} & & \end{array},$$

where $\tilde{k}$ is the number of trusted friends who think that *j* is a cheater.

Let us set the action of *i* based on this expectation, similar to above:

$$a_{i}=\{\begin{array}{ccc} \mathrm{cheat} & \mathrm{if} & e_{i,j}=\mathrm{cheater}\\ {\mathrm{type}}_{i} & \mathrm{if} & e_{i,j}=\mathrm{cooperator} \end{array}.$$

**Figure 2.**
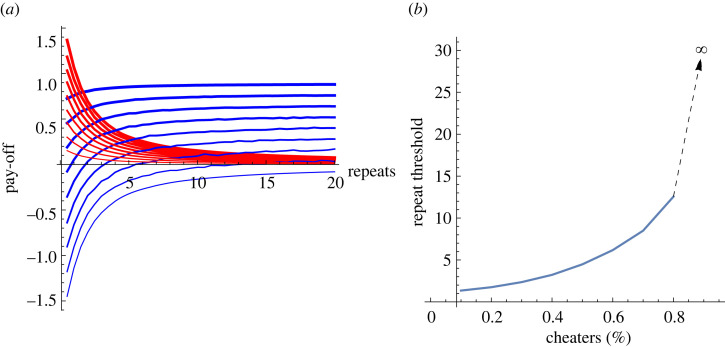
The interaction between repeat number, pay-offs and cooperation thresholds when the agents form a network and gossip. (For the definitions of the curves, see figure 1. Average of 1000 simulations. For each repeat, the network was randomly generated, with parameters *n* = 10 and *k* = 4.) The figure illustrates that introducing gossip does not change the basic pattern of the game: similar to the ‘plain vanilla’ case, cooperation is easier when interaction frequency is high and the cheater/cooperator ratio is low.

On the surface, the results are in a similar pattern to the previous, no gossip version (figure 2), although each agent has a stricter condition to cooperate.

**Figure 3.**
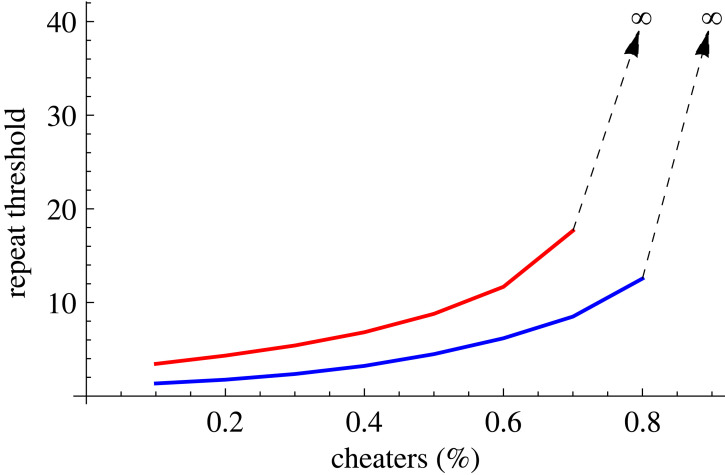
Cooperation threshold comparison between the ‘plain vanilla’ and ‘gossip-on-network’ cases. The red curve is identical to figure 1*b*, while the blue curve is the same as figure 2*b*. The figure illustrates that gossip speeds up the rise of cooperation, i.e. fewer repeats are needed, independent of the cheater/cooperator ratio.

However, comparing the two results illustrates why gossip matters for cooperation: faster flow of information reduces the interaction repeat threshold (figure 3).

**Figure 4.**
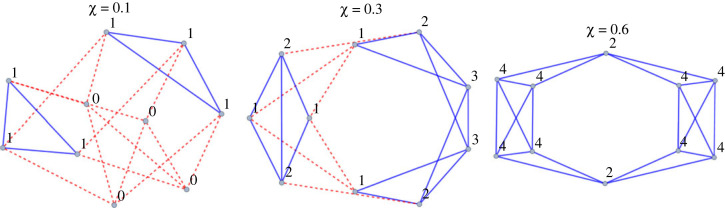
Illustration for variation in clustering coefficient: the same graph size and degree can correspond to substantially different graph structures. (All three graphs are 4-regular 10-sized: they have 10 nodes, with each of the nodes having four connections. The number next to each node is the number of closed triangles that node has, and *χ* is the average clustering coefficient. Blue lines: edges in closed triangles, red dotted lines: edges that are not part of a closed triangle.)

Thus, gossip allows faster identification of cheaters, and thus an earlier shift to a cooperative group.

### 2.3. The importance of network structure

Notice that in the gossip-on-network version above, the key to the improved cheater detection was that people who were connected to each other were able to pass on information, i.e. they gossiped and tracked others' reputation. By the nature of gossip as defined here, the possibility of gossip only matters if there is a shared connection between the two agents exchanging information about the third. As they are only interacting with those that are in their social network, such gossip can only take place if there is a closed network-triangle among them.

Given that we set the parameters at *n* = 10, and *k* = 4, it is known that there are 59 different non-isomorphic, connected graph structures, that is, there are 59 graphs that are different to each whichever direction you turn them [67]. There is considerable variation among them in terms of number of closed triangles (figure 4).

The average clustering coefficient, *χ*, of this set of 59 graphs ranges from 0% to 70%. (The clustering coefficient is the proportion of closed triangles compared to maximum possible triangles. That is, it is a measure of how interconnected the agents are, with both the size of the group and the number of ‘friends’ staying the same.)

**Figure 5.**
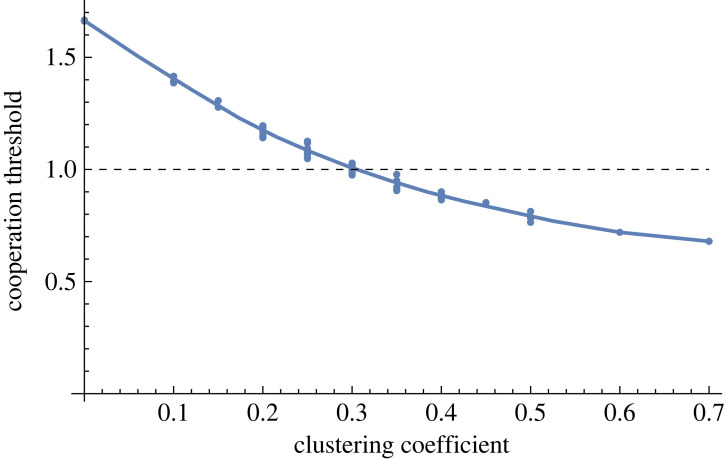
The clustering coefficient drives the differences in how easily cooperation emerges on graphs that all have 10 nodes, with each node having four connections. Each blue dot represents a different graph, the line is a regressed interpolation. (*x*-axis: average clustering coefficient of the graph, *y*-axis: threshold for average number of interactions above which the cooperative strategy is more advantageous than cheating. Number of cheaters: *m* = 1, randomly chosen in each new simulation, repeat number: 1000.)

I calculated the cooperation threshold for each graph in the set, for the example of *m* = 1. The results show that all else equal, the clustering coefficient drives the differences in cooperation among the graphs (figure 5). The downward sloping line shows that the higher the clustering coefficient is, the easier it is to maintain cooperation.

Notice that the threshold curve is not only downward sloping in figure 5, but it is also under 1 when the clustering coefficient is higher than 30%. Which means that, in this example, gossip can spread so fast that cheating becomes a loss-making strategy even before the cheater meets every one of its connections. (For cases in which there are more than one cheater, i.e. *m* > 1, see the electronic supplementary material, SM 2. Note: this paper's aim is to illustrate that structure matters for maintaining cooperative stance via gossip, and not for the original emergence of cooperation. Hence, we focus on the cases where gossip is already present, that is, the *m* values are low. The curves depicted in figure 5 and the electronic supplementary material, figure S1 are downward sloping for all meaningful *m*s. For high *m*s, the repeat threshold goes to infinity, figure 3, and hence the clustering coefficient-based differentiation becomes irrelevant.)

This result is an illustration for the interaction between the structure of the social network and cooperation. When the clustering coefficient is low (the ‘friends’ are not connected to each other), cooperation cannot be boosted by gossip. In these cases, if cooperation emerges, it is so owing to other mechanisms. Vice versa, when the clustering coefficient is high (the ‘friends’ are also ‘friends’ to each other), gossip makes maintaining cooperative stance among the group members easier. In other words, network structure matters when people track each other's reputation via gossip.

## 3. Discussion

The role of the clustering coefficient is central to the Structural Microfoundations Theory. This theory is based on the observation that, unlike most non-human networks, our species' social networks can have varied kinds of network edges, such as kinship, friendship and romantic relationships [68]. These edge types have different consequences for the local structure of the social network [56–58]. As demographic processes change the relative frequencies of the edge types, they can also lead to a change in the structure, in particular, in the clustering coefficient of the social network.

These processes, such as, falling fertility, urbanization, migration, wars and epidemics, have characterized our species’ past century, and together form a historically new framework for human behaviour. As these demographic processes cause a fall in the clustering coefficient, this triggers adaptive responses from the society. On one hand, these can be individual responses such as, increased norm violations, the rise of homophily based friendship, a shift to value fundamentalism and increased susceptibility to type-signalling institutions. On the other hand, the responses can be on the level of the society, such as, the rise of law, the emergence of kin-cue-using ideologies and the fake news industry. At the heart of these models is the assumption that, all else being equal, there is a causal relationship between the clustering coefficient and the level of cooperation in human societies. The model above spells out this mechanism.

Although the main goal of this paper is to provide an illustration for the underpinnings of the Structural Microfoundations Theory, by showing how the clustering mechanics of social trust works in the particular case of the *n*-sized *k*-regular connected graphs, it also introduces a small contribution to the literature. The effect of the clustering coefficient on cooperation stance in this particular graph family has not, to my knowledge, been shown before.

The main finding, i.e. that the higher the clustering coefficient the more cooperation, has some limitations. Reaching the maximum clustering coefficient may not be optimal for collective action. This might be the case, for instance, when there are parallel behaviours that affect the group via another mechanism [69], like teaching an ‘information value’ [70,71]. This observation has parallels with some of our earlier work on negative effects of social stratification, in which the loss of collective action efficiency came from a particular status dynamics [72].

There are two immediate future extensions.

The first has to do with the optimal way to formulate expectations about the future behaviour of others. There are many ways to form perceptions about reputation and respond to them, and the particular choice of reputation assessment method interacts with the chance and speed of the rise of cooperation [73]. It would be interesting to ask if the optimal reputation assessment technique is dependent on the clustering coefficient, and thus likely to shift with demographic processes. In particular, models of perspective taking, and sharing information about reputation thresholds [74] combined with the structural dynamics in the Microfoundations Theory could shed a new light on why public information about reputation may be likely to foster cooperation, especially in post-demographic transition societies.

Second, it would be intriguing to ask if some of the vast kaleidoscope of social technologies that human cultures invented in history are ways to increase the clustering coefficient. In particular, do institutions that regulate inequality [72], a social technology that is present in all human societies, increase social network interconnectedness? Is there an interaction between the rise of moral preferences [75,76] and network structure? Does the post-demographic transition drive towards highly clustered friendship groups make social network fragmentation more likely?

## Data Availability

I have uploaded the code in the form of a Mathematica notebook as the electronic supplementary material [77].
